# Squeezing the eggs to grow: The mechanobiology of mammalian folliculogenesis

**DOI:** 10.3389/fcell.2022.1038107

**Published:** 2022-12-02

**Authors:** Arikta Biswas, Boon Heng Ng, Vinod S/O Prabhakaran, Chii Jou Chan

**Affiliations:** ^1^ Mechanobiology Institute, National University of Singapore, Singapore, Singapore; ^2^ Department of Biological Sciences, National University of Singapore, Singapore, Singapore

**Keywords:** folliculogenesis, mechanobiology, oocyte, mechanotransduction, mammalian reproduction, theca cell

## Abstract

The formation of functional eggs (oocyte) in ovarian follicles is arguably one of the most important events in early mammalian development since the oocytes provide the bulk genetic and cytoplasmic materials for successful reproduction. While past studies have identified many genes that are critical to normal ovarian development and function, recent studies have highlighted the role of mechanical force in shaping folliculogenesis. In this review, we discuss the underlying mechanobiological principles and the force-generating cellular structures and extracellular matrix that control the various stages of follicle development. We also highlight emerging techniques that allow for the quantification of mechanical interactions and follicular dynamics during development, and propose new directions for future studies in the field. We hope this review will provide a timely and useful framework for future understanding of mechano-signalling pathways in reproductive biology and diseases.

## 1 Introduction

**FIGURE 1:**
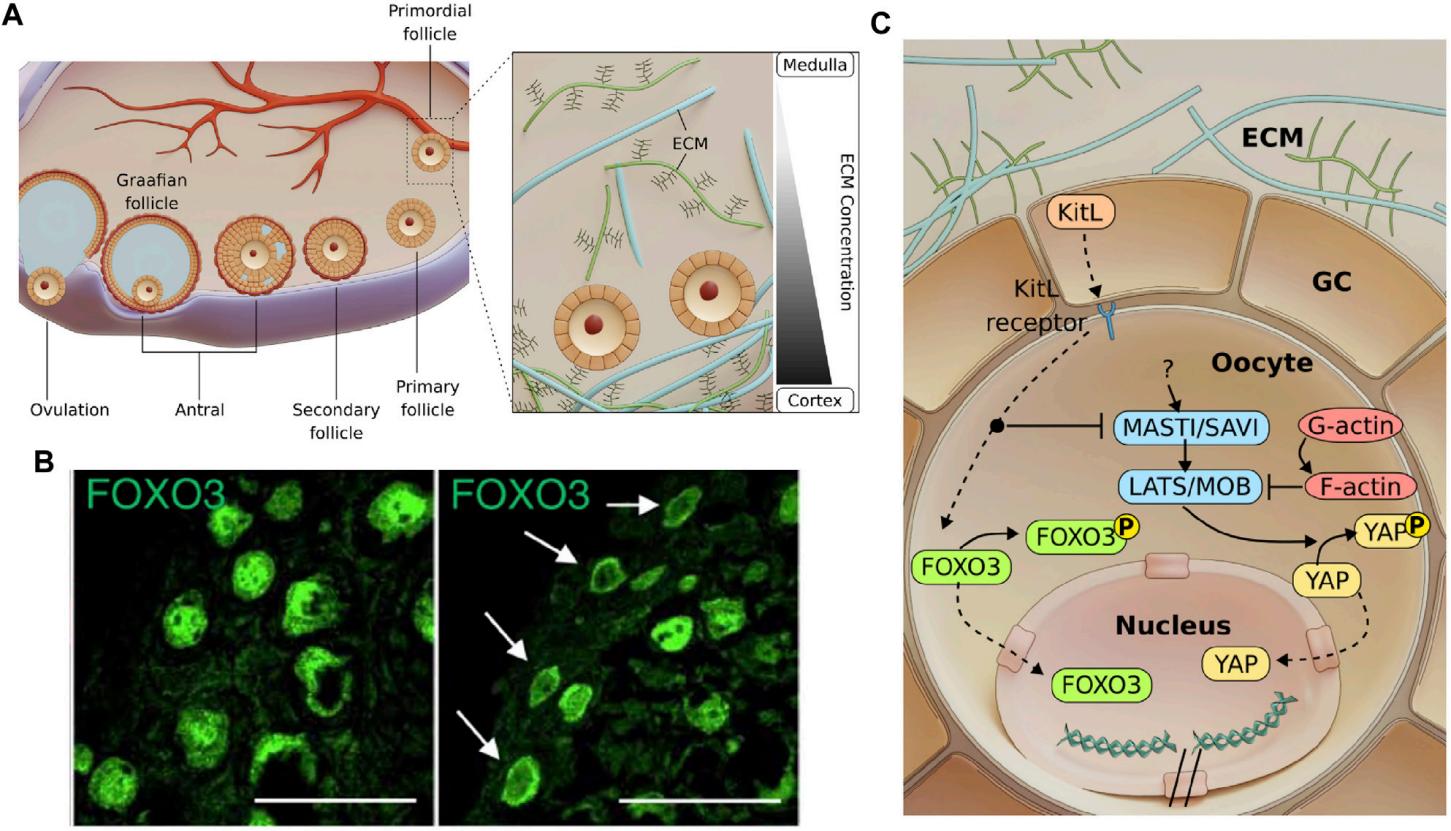
Follicle development and possible mechano-signalling pathways involved in primordial follicle activation. **(A)**. Mammalian folliculogenesis is classified into the primordial, primary, secondary and antral follicle stage. Antral follicles undergo ovulation to release the cumulus-oocyte complex before forming a corpus luteum. Inset: A gradient of ECM stiffness was proposed to extend from the soft ovarian medulla to the stiffer cortex where the dormant primordial follicles predominantly reside. **(B)**. Images showing that primordial follicles can be activated *via* ECM degradation (right), as indicated by FOXO3 cytoplasmic localisation compared to the nuclear localisation of FOXO3 in dormant primordial follicles (left). Scale bar: 50 μm. Images from (Nagamatsu et al., 2019). **(C)**. Mechanosensitive Akt and Hippo pathways govern primordial follicle activation. GCs express Kit ligands (KitL), which are bound to the Kit receptors on the oocyte. Activating the Akt pathway leads to phosphorylation of FOXO3 in the cytoplasm. The Akt pathway also disrupts the Hippo pathway at MST1 to allow for YAP nuclear localisation and downstream activation and growth of follicle. Mechanical stress exerted by the GCs may independently activate mechanosensitive proteins and contribute to dormancy.

In mammals, the ovarian follicles housing the oocytes are the functional units for female reproduction. The maturation of follicles, or folliculogenesis, is not only essential for supporting oogenesis, but also triggers the production of hormones for female sexual characteristics and early pregnancy. The developmental stages of follicles, loosely defined by the follicle size, the number of cells at the follicular envelope and the morphology, can be classified into the primordial, primary, secondary and antral follicle stage (Hertig and Adams, 1967) (Figure 1A). Folliculogenesis begins with the primordial follicle stage, which consists of an oocyte surrounded by a single layer of somatic granulosa cells (GCs). Upon activation, the primordial follicles develop into the primary follicles, which are characterised by the formation of cuboidal-shaped GCs and the zona pellucida (ZP) that encapsulates the oocyte while maintaining transzonal projections between the oocyte and the GCs. These primary follicles then grow into secondary follicles characterised by multiple layers of GCs and an outer layer of theca cells (TCs). Multiple pockets of fluid-filled lumens form within the GCs as the secondary follicles increase in size and acquire stratified layers of GCs and TCs. These lumen eventually resolve into a single antrum that grows in size. Under the action of luteinizing hormone, the theca wall undergoes extensive extracellular matrix (ECM) remodelling, which, together with antrum expansion, lead to follicle rupture and the release of oocytes out of the ovary, a process known as ovulation.

Early work on ovarian folliculogenesis was largely descriptive, relying on histology, histochemistry and electron microscopy (Hertig and Adams, 1967). There were extensive functional studies using molecular genetics and transgenics approaches, which revealed key genes and proteins that are required for primary follicle activation and growth (Choi and Rajkovic, 2006; França and Mendonca, 2022). The advancement of immunofluorescence imaging further enables the visualisation of follicular cells and the cytoskeletal proteins during development (Da Silva-Buttkus et al., 2008; Mora et al., 2012), paving the way for a more quantitative approach to understand follicular morphogenesis. Along with these studies, novel approaches were developed to culture follicles *ex vivo*, such as the seminal work by Eppig *et al.* on 2D systems and organ culture (Eppig, 1977; Eppig and Schroeder, 1989; Eppig and O'Brien, 1996). In the 2000s, a series of works on culturing follicles in 3D using novel biomaterials were introduced (Pangas et al., 2003; Xu et al., 2006a; West et al., 2007; Hornick et al., 2012), which provide a more native follicle environment with tunable stiffness for optimal follicle growth and survival.

While these studies revealed key paracrine and autocrine signalling patterns in the mammalian ovary, the biomechanics and the roles of mechanical signalling during folliculogenesis have not been investigated as much. It is known that mechanical forces play an integral role in controlling cellular dynamics and functions in development, tissue homeostasis and cancer growth (Heisenberg and Bellaïche, 2013; Hannezo and Heisenberg, 2019). Effective coordination of long-range force transmission and mechanosensing of cells do not only lead to robust tissue morphogenesis, they also impact cell fate specification and tissue patterning *via* mechanotransduction (Chan et al., 2017). In recent years, new evidence has emerged showing that mechanical cues play a critical role in regulating ovarian folliculogenesis. *Ex vivo* culture of follicles revealed that the follicle growth is sensitive to the surrounding matrix stiffness, and compressive stress exerted by the matrix can act against follicle expansion and development. Fragmentation of the ovary, which releases tissue mechanical tension and disrupts F-actin, can promote follicle activation and growth *via* Hippo signalling pathway, a well known mechanotransduction pathway (Kawamura et al., 2013). It has also been hypothesised that the regional difference in ECM stiffness at the outer cortex and the inner medulla may contribute to differential follicle growth or migration (Woodruff and Shea, 2011), which was supported by recent studies on primate (rhesus monkey) and bovine ovaries (Hornick et al., 2012; Henning and Laronda, 2021). These studies highlight the importance of applying tissue mechanics to understand how the follicle size, number and positioning are controlled robustly during ovarian development.

Follicle development is often associated with extensive tissue remodelling (Smith et al., 1999), and changes in tissue mechanical properties have been implicated to influence oocyte quality and fertility. Indeed, ovarian ageing is highly correlated with increased fibrosis and tissue stiffness (Amargant et al., 2020) due to increased expression or altered composition of ECM in the stroma (Briley et al., 2016). Interestingly, ovarian diseases such as polycystic ovarian syndrome (PCOS) (Wood et al., 2015) have densely collagenized and thickened cortex, and are characterised by anovulation, probably due to the lack of ECM degradation or abnormal ECM architecture. Similarly, endometriosis was shown to be associated with abnormal mechanical rigidity in the ECM (Huang et al., 2022). These studies suggest that proper follicle growth requires a biomechanically permissive environment, and calls for a need to better understand the mechanobiological principles governing folliculogenesis.

In this review, we first introduce the various stages of ovarian development and the classical molecular signalling pathways associated with each stage. We then discuss how mechanical stress may be generated by cells within the follicle, and the ECM and stromal cells at the extra-follicular level to impact follicle activation, growth and tissue patterning during ovarian development. We will also introduce the relevant biophysical techniques and reconstitutive biomimetic approaches that allow us to better investigate the underlying mechano-signalling pathways regulating folliculogenesis. Finally, we provide some future perspectives and highlight open questions for the field of ovarian mechanobiology.

## 2 Primordial follicle dormancy and activation

Prior to the primordial follicle formation, the female primordial germ cells first form cell cysts during embryonic development. In mouse, following birth, cyst breakdown occurs due to the invading GCs, which eventually surround the germ cells to form dormant primordial follicles (Lei and Spradling, 2013; Lei and Spradling, 2016). Bidirectional communication between the oocyte and the GCs has been shown to be essential at this stage, as the absence of either the oocyte or the GCs lead to follicle death (Eppig, 1979). The oocyte controls glycolysis within the GCs *via* the expression levels of glycolytic genes (Sugiura et al., 2005) and influences the level of luteinizing hormone receptor (LHR) in the GCs (Eppig et al., 1997). The GCs, on the other hand, play an active role in modulating the transcriptional activity and chromatin remodelling of the oocyte (De La Fuente and Eppig, 2001), and express Kit ligand (KitL) which binds to the c-Kit receptors on the oocyte to initiate AKT-mediated follicle activation (Jones and Pepling, 2013). The activation of dormant primordial follicles may also involve protein interactions from within the cell or across cell-cell junctions. In this section, we focus on the PI3K/Akt and Hippo pathways, two signalling pathways that have been identified as major regulators of primordial follicle activation. These pathways may be triggered by extrinsic mechanical stress imposed by the surrounding GCs and the ECM (Figure 1A, inset), which helps to maintain primordial follicle dormancy and ensure sufficient ovarian follicle reserve throughout the fertility period.

The Akt pathway is a well-established signalling pathway that responds to extracellular signals and promotes growth and survival (Hemmings and Restuccia, 2012). In response to a KitL, KIT proto-oncogene receptor tyrosine kinases (c-Kit) induce a kinase cascade, which in turn phosphorylates and activates AKT. Phosphorylated AKT then phosphorylates the transcription factor FOXO3 (Forkhead Box O3), leading to cytoplasmic localisation of FOXO3. It was demonstrated that FOXO3 nuclear export coincides with primordial follicle activation (John et al., 2008) and FOXO3 overexpression leads to increased reproductive capability in mice (Pelosi et al., 2013). Constitutively active FOXO3 was found to cause infertility (Liu et al., 2007), suggesting that nuclear FOXO3 is essential for dormancy regulation but only at a sufficient level. In contrast, the lack of FOXO3 resulted in uncontrolled follicle activation and early infertility (Castrillon et al., 2003), but did not affect follicular growth after activation (John et al., 2007). This suggests that FOXO3 specifically regulates the primordial follicle dormancy, and can be used as a reliable marker for primordial follicle activation.

Primordial follicles are mainly found at the cortex of the ovary, surrounded by abundant ECM. Recent work by Nagamatsu *et. al.* demonstrated that the cortical ECM of mouse ovaries provides mechanical stress to maintain the primordial follicles in their dormant state (Nagamatsu et al., 2019) (Figure 1B). In this study, they showed that the disruption of collagen leads to FOXO3 translocation to the cytoplasm in primordial follicles, while application of external pressure restores nuclear FOXO3 and dormancy. Notably, primordial follicles that are under mechanical stress exhibit nuclear rotation. However, the mechanosensing mechanism for FOXO3 dynamics and how forces are related to nuclear rotation remains unknown. Of note, the oocytes expanded in size upon ECM abolishment, suggesting that they are physically compressed in their dormant state. The transient change in oocyte volume also implies fluid exchange between the oocyte and the surrounding GCs and possible volume regulation by hydraulic stress.

Apart from the Akt pathway, the Hippo pathway is another well known mechano-signalling pathway. The Hippo pathway can respond to various signals, such as ECM stiffness (Dupont et al., 2011), substrate stiffness (Thomasy et al., 2013), stretching (Aragona et al., 2013), cell geometry (Dupont et al., 2011), cell density (Zhao et al., 2007), cellular tension (Perez Gonzalez et al., 2018) and shear stress (Lee et al., 2017). These factors were integrated by the Hippo pathway to regulate various cellular processes such as growth, development and homeostasis (Wu and Guan, 2021). When the Hippo pathway is activated, the Yes-associated protein (YAP) becomes phosphorylated and localises to the cytoplasm, where it remains non-functional or gets degraded. However, when the Hippo pathway is disrupted, non-phosphorylated YAP translocates to the nucleus to form a transcription activation complex with TEA domain family members (TEAD 1-4), leading to expression of downstream genes. The Hippo pathway was found to play a role in follicle growth. Disruption of the Hippo pathway caused a primary ovarian insufficiency (POI)-like phenotype (St John et al., 1999), indicating that YAP might be necessary for follicle activation. This is further corroborated by a study of Kawamura *et al.*, who demonstrated that ovarian fragmentation can lead to F-actin polymerisation, increased nuclear YAP localisation and primordial follicle activation (Kawamura et al., 2013). Moreover, F-actin polymerisation itself leads to increased nuclear YAP localisation and follicle growth (Cheng et al., 2015), highlighting YAP’s mechanosensing capability and its role in follicle activation.

Given that the dormant oocytes with nuclear FOXO3 are mechanically compressed (Nagamatsu et al., 2019), and that compressive stress is implicated in YAP signalling, this suggests a potential crosstalk between Akt pathway and Hippo pathway. There is evidence that the Hippo pathway functions downstream of the Akt pathway. In *Drosophila*, Borreguero-Muñoz et al. showed that AKT can directly disrupt the Hippo pathway (Figure 1C) (Borreguero-Muñoz et al., 2019). In mouse ovaries, the use of MK2206 (AKT inhibitor) not only suppressed follicle activation, but also led to an increase in pYAP-to-YAP ratio (Hu et al., 2019). This implies that AKT can disrupt Hippo signalling and trigger YAP nuclear localisation and follicle activation. This evidence points to the existence of a crosstalk between the Akt and Hippo pathways and should be carefully examined together in the context of primordial follicle activation.

The GCs surrounding the oocyte may also directly regulate oocyte functions through mechanotransduction (Figure 1C). Notably, the squamous GCs exhibit contractile stress fibres at the primordial follicle stage (Nagamatsu et al., 2019), and have flat morphology compared to the cuboidal GCs at the primary follicle stage. Is this change in morphology during activation a result of reduced compressive stress from ECM remodelling, or due to intrinsic change in GC contractility? Furthermore, since flat cells are often associated with high surface tension (Lecuit and Lenne, 2007), does it imply that the squamous GCs exert significant forces on the oocyte during dormancy, and the release of mechanical tension leads to follicle activation? Another open question is whether the GC mechanics can directly trigger the expression of KitL and downstream AKT-mediated activation in the oocyte. These questions will likely be addressed in the near future with the application of novel biophysical tools and *ex vivo* reconstitution approaches (see Section 6).

## 3 Mechano-signalling in pre-antral follicle development

**FIGURE 2:**
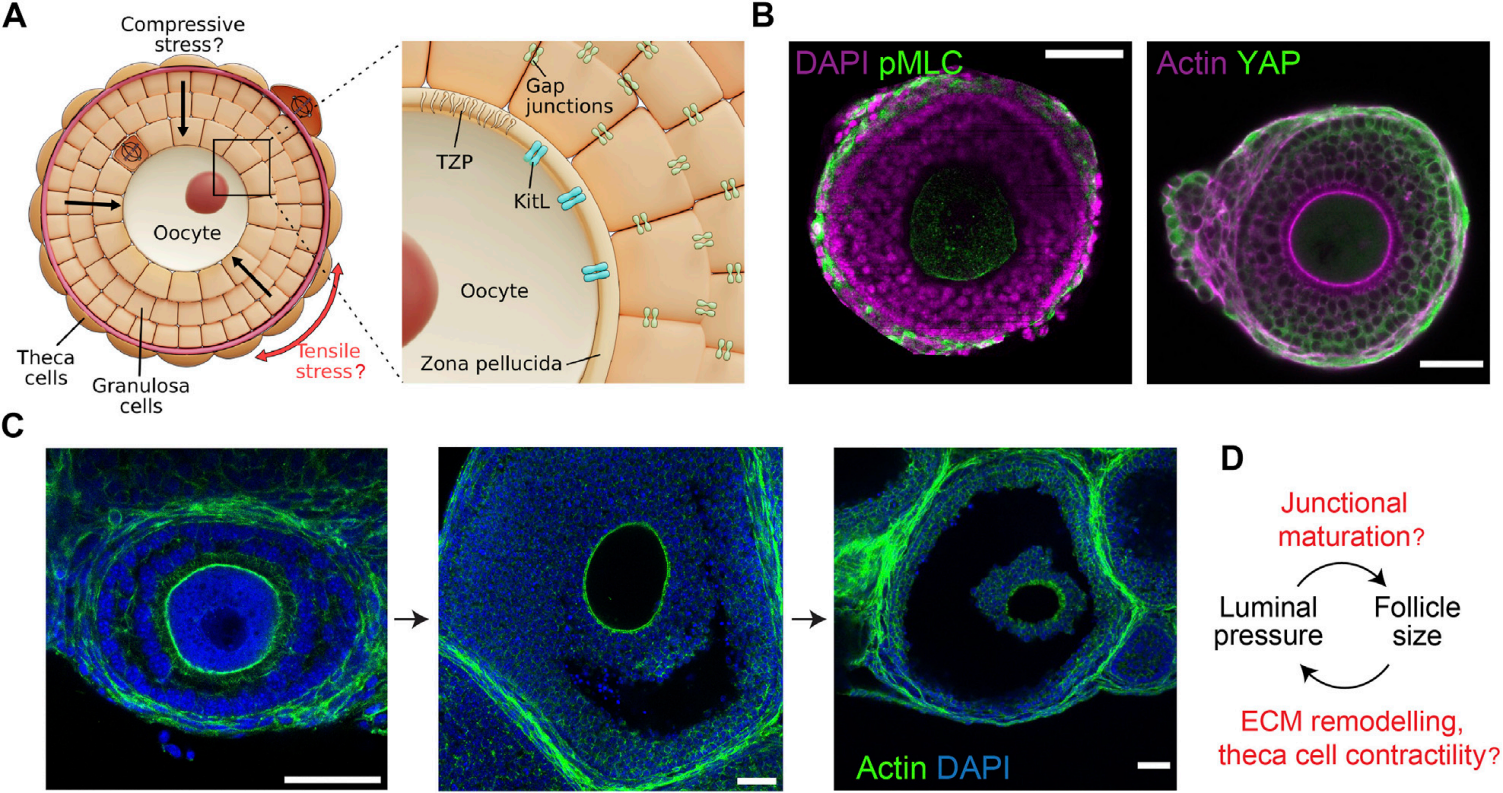
Mechano-hydraulic control of secondary and antral follicle development. **(A)**. During secondary follicle development, spindle-shaped TCs form around the basement membrane (dark red) and the GCs, potentially generating compressive stress within the follicle. Anisotropic stress pattern may guide cell division orientation in the TCs (circumferential) and the GCs (radial). Inset: Compressive stress may affect oocyte growth by modifying the oocyte-granulosa interactions mediated by the TZP and Kit ligands. Mechanical stress may also influence the GC proliferation *via* gap junction activities. **(B)**. Left: Isolated follicle immuno-stained for nuclei (DAPI) and phosphorylated-myosin light chain (pMLC), showing contractile TCs. Right: Follicle immuno-stained for actin and YAP, showing YAP nuclear localisation in the TCs only. Scale bar: 50 µm. **(C)**. Antral follicles immuno-stained for nuclei (DAPI) and actin. Luminal fluid accumulates at the intercellular space, eventually merging into a singly-resolved antrum that continues to expand till ovulation. Scale bar: 40 µm. **(D)**. Potential feedback loop between luminal pressure and follicle size: increased luminal pressure may trigger junctional maturation and follicle expansion through mechanosensing, while TC stiffening may control the final size of the follicles.

Secondary follicle development is characterised by the development of the spindle shaped TCs overlying the basement membrane (BM) around the GCs (Figure 2A). It takes more than 30 days for primordial follicles to become secondary follicles in rat ovaries, and 120 days in the case of human ovaries (McGee and Hsueh, 2000). Sizes of secondary follicles range from 59 to 303 µm in diameter as measured across multiple species (Griffin et al., 2006). Secondary folliculogenesis is also marked by a rapid increase in oocyte volume, although the oocyte is observed to remain in meiotic arrest (DiLuigi et al., 2008). The ZP surrounding the oocyte becomes thicker and more prominent at this stage for most organisms (Albertini et al., 2001). Here we focus our discussion on three key components in secondary follicles that may potentially regulate their development *via* mechanical signalling.

### 3.1 Basement membrane

One of the most crucial internal components of the follicle is the BM. The basal lamina separating the TCs from the GCs is typically composed of isoforms of collagen IV and laminin (O’Shea et al., 1978), but the relative compositions change during folliculogenesis (Rodgers et al., 2003; Berkholtz et al., 2006). Fibronectin is not prominent in the BM of primordial and primary follicles, but its expression becomes stronger at the secondary follicle stage (Akkoyunlu et al., 2003; Heeren et al., 2015). BM is nano-porous and can allow size-dependent passage of molecules across it. Molecules with molecular weights lower than 100 kDa can move freely through the BM, while those larger than 500 kDa cannot (Perloff et al., 1954; Shalgi et al., 1973). There exist reports on the pore size and thickness of BM in non-ovarian models (Abrams et al., 2000; Nicholas and Jacques, 2005; Halfter et al., 2013; Gaiko-Shcherbak et al., 2015), but these parameters have not been characterised in ovarian follicles. It is suggested that the composition, geometry, and crosslinking of the different proteins can dictate the BM mechanical properties (Miller, 2017; Ramos-Lewis and Page-McCaw, 2019), but the physical properties and mechanical functions of BM in folliculogenesis remain poorly characterised.

BM is common in most tissues in the body and is involved in a variety of diseases (Christensen et al., 2015). The thickness of the reticular BM (Saglani et al., 2006) and capillary BM (Ganda et al., 1983) has been implicated in asthma and diabetes respectively. It has been shown in epithelial cancers that cells can generate physical forces to invade the BM, leading to metastasis (Chang and Chaudhuri, 2019). Cancer-associated fibroblasts, myoepithelial cells and immune cells have also been implicated to regulate cell invasion. It is possible that similar conditions may apply to ovarian cancers. Hence, a deeper understanding of BM mechanics may inspire new approaches to modify the BM and help to prevent cancer progression.

The stiffness of the BM provides another key mechanical cue that may impact follicle development. The values of BM stiffness vary, ranging from ∼55 kPa in mouse mesentery to 400-3,000 kPa in mouse renal tubules (Bhave et al., 2017; Glentis et al., 2017). In *Drosophila*, BM stiffness has been shown to determine the shape of developing egg chambers (Crest et al., 2017; Chen et al., 2019) and in altering cell migration *in vivo* in the central nervous system (Kim et al., 2014; Sánchez-Sánchez et al., 2017). Softer BM in the *Drosophila* wing disc allowed the wing disc to expand and flatten (Ramos-Lewis and Page-McCaw, 2019). Increased niche-derived mechanical stress due to increased BM stiffness in aged murine hair follicle stem cells has been observed to repress transcription, silence bivalent promoters and compromise stem cell potential (Koester et al., 2021). Recently it has also been shown that the intricate interplay between cell-cell and cell-BM interactions can contribute to stratified epithelial budding and branching morphogenesis of murine embryonic salivary glands (Wang et al., 2021).

In ovarian development, the BM has been suggested to protect ovarian follicles from physical damage (Figueiredo et al., 1995). It was proposed that the stiff basal lamina acts as a mechanical barrier for the proliferation and inward division of GCs (Da Silva-Buttkus et al., 2008): the GCs become more densely packed when they divide in a radial fashion within the follicles, thereby leading to the emergence of multi-layered GCs at this stage (Da Silva-Buttkus et al., 2008). Here we speculate that as the follicles grow in size, the BM may deform or undergo active remodelling to accommodate for the growing number of GCs contained within. How this changes the BM mechanical properties and regulates TC and GC functions will constitute an exciting topic for future research.

### 3.2 Granulosa cells

The internal geometry of a follicle loosely resembles that of a multicellular spheroid and it is plausible that mechanical cues affecting spheroid growth could also play a role in folliculogenesis. Increased mechanical stress inside tumour spheroids have been shown to lead to reduced cellular proliferation (Delarue et al., 2014; Dolega et al., 2017). As the spheroids grow, compressive stress can build up and promote cancer cell migration (Tse et al., 2012). It has been shown that the tumour spheroid growth responds to the stiffer mechano-environment through cytoskeletal remodelling and ROCK signalling pathway (Taubenberger et al., 2019). In the developing wing imaginal disc of *Drosophila*, the varied stress patterns between cells at the periphery (tension) and the cells in the centre (compression) lead to differential cell shape and division orientation (LeGoff et al., 2013). In secondary follicles, a similar mechanism may operate where follicle growth leads to the emergence of anisotropic stress pattern, characterised by radial compressive stress in the GC layer and tangential tensile stress at the theca layer. This may in turn direct cell division pattern and follicle morphogenesis (Figure 2A).

GCs secrete mucopolysaccharides which thicken and rigidify the ZP during follicle growth, thereby changing the mechanical environment around the oocytes (Ouni et al., 2020). Transzonal projections (TZPs) are hair-like projections from the GCs which invade the ZP and maintain direct contact with the oocyte (El-Hayek et al., 2018). The communication between the oocyte and its surrounding GCs is also mediated by connexin 37, a gap junction protein that is present at the tip of the TZPs (Simon et al., 1997; Ackert et al., 2001). Among the GCs, it is known that another type of gap junction proteins, connexin 43, connect the GCs and help with the exchange of nutrients like amino acids, glucose, ions, and cGMP in order to maintain cellular osmolarity and metabolism (Eppig, 1991; Winterhager and Kidder, 2015). Gene knockout studies have shown that connexin 37 are not only essential for oocyte maturation, they are also required for the luteinisation of GCs prior to ovulation (Simon et al., 1997). Loss of connexin 43 in mutant ovaries showed impaired GC proliferation, absence of antrum and developmentally incompetent oocytes (Ackert et al., 2001). Connexins in endothelial cells are known to be affected by shear stress and connexin 43 hemichannels in osteocytes are known to be opened by mechanical loading which helps in bone formation (Islam and Steward, 2019; Riquelme et al., 2020; Zhao et al., 2020). Whether connexins respond to mechanical stimuli and regulate follicle growth requires further investigation.

As the GCs become multi-layered, several signalling molecules–namely, BMP-15 and GDF-9—secreted by the oocyte start to impact this process (Dong et al., 1996; Moore et al., 2003). As described in the previous section, both these factors along with the Kit ligands and their receptors are believed to take part in a feedback loop between the oocyte and the GCs during this developmental stage (Eppig, 2001; Otsuka and Shimasaki, 2002). A similar bidirectional communication also exists between the GCs and TCs. While the Kit ligands and other growth factors from the GCs can prompt TC proliferation (Huang et al., 2001; Matsuura et al., 2002; Field et al., 2014; Shiomi-Sugaya et al., 2015), several BMPs secreted by the TCs are shown to impact GC proliferation (Lee et al., 2001; Glister et al., 2005) through the Wnt (Wang et al., 2010), Notch (Trombly et al., 2009), and Hedgehog (Wijgerde et al., 2005) signalling pathways. Though some of the signalling pathways have been implicated in tissue mechanics in non-ovarian developmental systems (Bonewald and Johnson, 2008; Brunt et al., 2017; Stassen et al., 2020; Yang et al., 2021), definitive evidence on potential mechano-regulation of these signalling pathways in the ovary remains to be established.

### 3.3 Theca cells

TCs are observed to appear at the periphery of secondary follicles that have at least two layers of GCs (Figure 2A) (Magoffin and Weitsman, 1994), and the process is gonadotropin independent. However, the origin of these cells remains debatable. While some believe that they originate from putative stem cells in ovaries (Honda et al., 2007), others suggest that they are derived from progenitor cells at the embryonic stage (Liu et al., 2015). Though not characterised, it is hypothesised that the cells are recruited from the ovarian stroma mediated by oocyte or GC secreted factors (Orisaka et al., 2009). TCs provide structural integrity to the follicles and produce important endocrine regulatory factors like androgens and other growth regulatory factors (Erickson et al., 1985; Young and McNeilly, 2010). Malfunction of TCs such as overproduction of TC-secreted androgens leads to PCOS (Azziz et al., 2009; Huang et al., 2010), which is a leading cause of female infertility that affects 4-20% of reproductively aged women worldwide (Deswal et al., 2020).

The theca layer is not homogeneous. The layer consists of the inner cells bordering the BM, also known as the *theca interna*, which secrete steroids that serve as substrates for estrogen production from the GCs (Magoffin et al., 1995). The outermost layers of TCs, the *theca externa*, are fibroblast-like in nature. They emerge at the later stages of follicle development, and are believed to play a role during ovulation. During follicle expansion in this growth phase, the theca layers are known to provide nutrients by developing vasculature through secreting vascular endothelial growth factor (VEGF) (Jones and Shikanov, 2019), thereby leading to increased oxygenation in the follicle (Shin et al., 2005).

**FIGURE 3:**
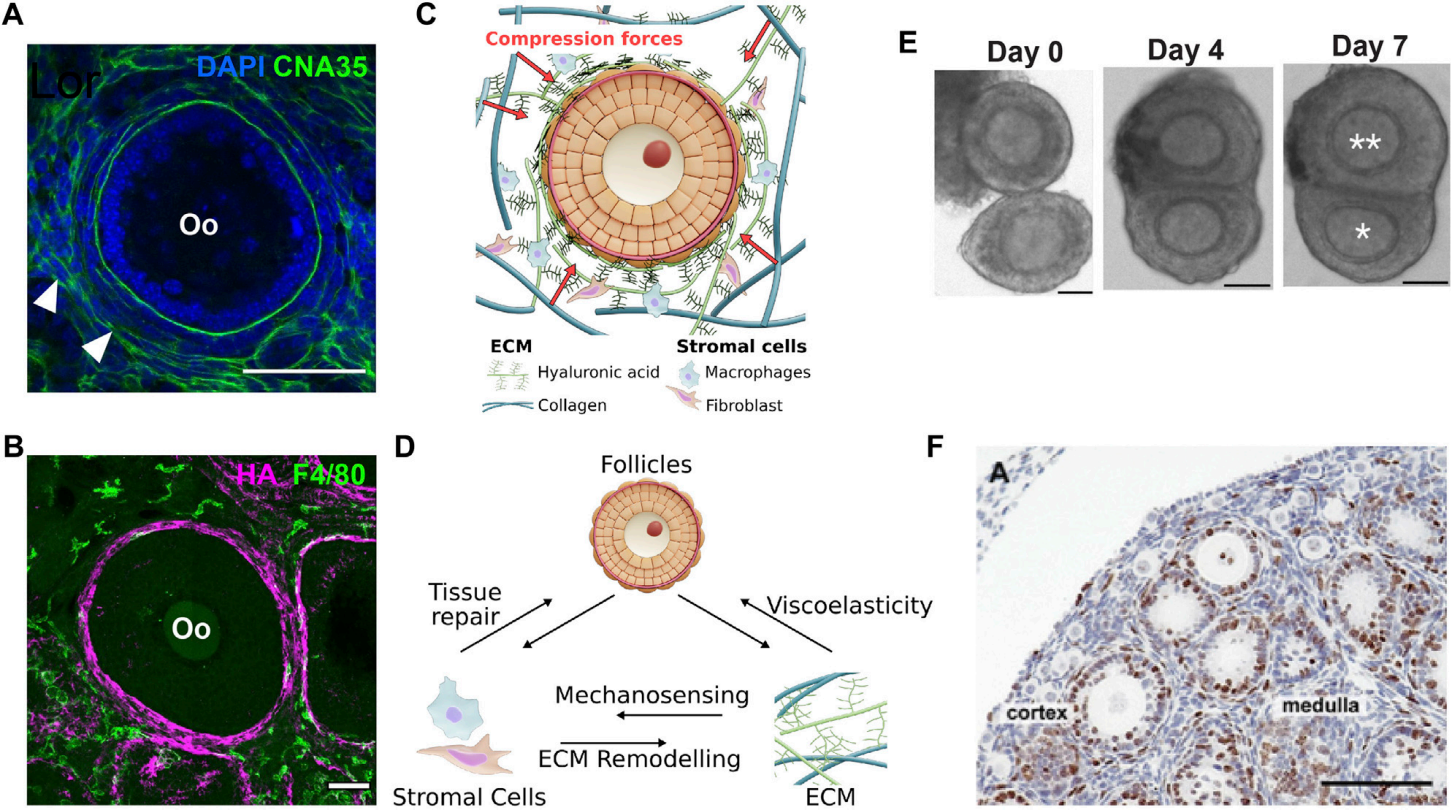
Stromal mechanics and inter-follicle dynamics in ovary development. **(A)**. Labelling of CNA35, a collagen-binding protein, shows the presence of basement membrane within the follicle and at the interstitial tissue (white arrowhead). Scale bar: 50 μm. **(B)**. Macrophages labelled with F4/80 were observed at the interstitial matrix, while the hyaluronic acid (HA) was highly expressed at the TC layer. Scale bar: 50 μm. **(C)**. Growing follicles may be mechanically confined by the surrounding ECM that comprises mainly of collagen and HA. **(D)**. Potential feedback interactions between stromal cells, ECM and follicle growth during ovarian development and ageing. Changes in ECM composition and mechanical properties may activate immune response from macrophages *via* mechanosensing pathways, or the stromal cells may actively remodel the ECM or participate in tissue repair during follicle death or post-ovulation. Changes in ECM viscoelastic properties may directly influence follicle growth. (**E–F)**. Follicle-follicle interactions may provide additional mechanical signals to influence follicle growth. **(E)**. *In vitro* culture of follicle doublet shows that the follicles can merge over the course of 7 days to generate a dominant, growing follicle (double asterisks) and a ”subordinate” follicle with limited growth (single asterisk). Scale bar: 50 μm. **(F)**. Evidence of close contact among pre-antral follicles in a P12 mouse ovary. Scale bar: 100 μm. Image from (Da Silva-Buttkus et al., 2008).

While the role of TCs in steroid production and hormonal regulation is well documented, the structural and mechanical functions of these cells are less well understood. Recently, cancer-associated fibroblasts have been shown to self-organise to form a capsule around tumour cells and initiate mechanotransduction through active compression (Barbazan et al., 2021). The contractile TCs may wrap around the follicles in a similar fashion and provide compressive stress to coordinate signalling and growth within the follicle (Figure 2B). A recent study revealed the presence of mechanical heterogeneity within the follicle, where the TCs were shown to have a higher loss tangent (>20%) than the GCs, particularly during the antral follicle stage (Chan et al., 2021). The loss tangent here can be understood as the ratio between the cell’s effective micro-viscosity and stiffness. A higher loss tangent of TCs therefore indicates greater energy dissipation and a more viscous response when subjected to mechanical stress. This may be associated with the increased hyaluronan production by the TCs (Figure 3B) (Amargant et al., 2020).

Despite growing evidence that mechanical signalling can impact follicle development, questions remain on how these mechanical forces are generated and maintained within the follicles. In particular, it is not known how GC proliferation, which may generate tissue pressure, is coupled to BM remodelling and TC-generated compressive stress during follicle development. Addressing this will require novel methods to quantify cellular dynamics, tissue mechanics and mechanosensing activities in the ovarian follicles (see Section 6).

## 4 Tissue hydraulics during antral follicle development and ovulation

A key morphological event is the emergence of a fluid-filled cavity (lumen) in the antral follicles (Figure 2C). Fluid cavity can generate hydrostatic pressure that can modulate cell-cell junctional remodelling or ion pump activities that in turn leads to organ or embryo size control and tissue patterning (Chan et al., 2019; Mosaliganti et al., 2019). A similar feedback mechanism may operate during antral follicle morphogenesis (Figure 2D). In addition, the lumen can act as a signalling hub to drive cell differentiation and cell shape changes around the lumen (Durdu et al., 2014; Ryan et al., 2019). In mammalian species, the antrum size varies from species to species: larger species such as human and bovine have larger follicles with the fluid comprising a substantial volume fraction (>95%) of the follicles at ovulation (Rodgers et al., 2001), while follicles of smaller species such as rats and mice contain less follicular fluid (Rodgers and Irving-Rodgers, 2010). Despite previous reports showing a correlation between antrum formation and oocyte maturation (Dumesic et al., 2015), the potential mechano-chemical functions of antrum in folliculogenesis remain enigmatic. Similarly, there remains limited studies on how the micro-lumen first emerge and coalesce into a single antrum, which is essential for late ovulation. One likely mechanism is osmotic regulation, where the antrum fluid could be sourced externally from the blood vessels formed around the TCs that encapsulate the antral follicles. Since the GCs lack functional tight junctions (Mora et al., 2012), it would not be possible to establish an osmotic gradient across the membrane granulosa with small ions like sodium. Instead, larger molecules such as hyaluronan and proteoglycans appeared to be trapped in the antrum which may generate a significant osmotic gradient to draw in fluid from the theca capillary (Clarke et al., 2006; Rodgers and Irving-Rodgers, 2010). However it remains unclear how the GCs actively secrete osmotically active molecules into the antrum, and if this is triggered by some signalling molecules released by the oocyte. One possible hypothesis is that some GCs within the follicle undergo apoptosis and are eliminated, thereby creating a hollow space that is filled with fluid. This process is also known as ‘cavitation’ (Sigurbjörnsdóttir et al., 2014). Cell death releases DNA fragments which, by virtue of their high molecular weights, may generate strong osmotic force to drive lumen expansion and coalescence (Clarke et al., 2006).

As mentioned, the GCs lack functional tight junctions but are connected by the gap junctions (Mora et al., 2012). This suggests that antrum development is not likely mediated by directed fluid transport through apico-basal polarity establishment, a mechanism widely reported in epithelial tissues and *in vitro* (Roignot et al., 2013). It is however worth noting that fluid accumulation can occur at the basolateral compartment through exocytosis or programmed cell shape changes (Schliffka and Maître, 2019). For example, during mouse blastocyst development, cytoplasmic vesicles are secreted and transported into intercellular space, leading to fluid accumulation at the onset of blastocoel formation (Ryan et al., 2019). During zebrafish gastrulation, mitotic rounding during cell divisions drive cell-cell contact disassembly in the deep cells, leading to increased interstitial fluid that translates to global tissue fluidization at the animal pole (Petridou et al., 2019). Whether these mechanisms operate during antral folliculogenesis constitute exciting topics for future research.

As the follicle matures into the Graafian follicle, it undergoes ovulation, which is characterised by a series of biochemical and morphological events that lead to its ultimate rupture and discharge of the mature oocyte out of the ovary. While it is known that elevated levels of luteinizing and follicle-stimulating hormones are required for ovulation, the precise mechanisms underlying this process remain unknown. Early studies pointed to both mechanical and enzymatic activities in follicle rupture during ovulation. Biophysical studies of follicle wall tension showed a large increase in wall extensibility prior to follicle rupture (Espey and Lipner, 1963), which is associated with the thinning of the theca wall due to ECM degradation by multiple proteolytic enzymes such as matrix metalloproteinases and plasmin (Curry and Smith, 2006). Interestingly, endothelin was proposed as a mechanotransduction gene that may facilitate follicular rupture and ovulation by binding to the smooth muscle at the theca externa, thereby leading to the contractile activity necessary for follicular rupture (Ko et al., 2006). Early studies on antrum fluid mechanics revealed no significant change in the hydrostatic pressure during antrum development (Espey and Lipner, 1963; Rondell, 1964; Bronson et al., 1979), potentially due to species difference and technical limitations. However, recent studies using servo-null micropressure system detects more than 40% increase in luminal pressure from preovulatory to late ovulatory phase in rat ovaries after human chorionic gonadotropin (hCG) stimulation (Matousek et al., 2001). Future studies leveraging on *ex vivo* culture and live imaging will provide better spatiotemporal dynamics of ovulation and will help to address how coordinated changes in lumen pressure, tissue mechanics and ECM remodelling lead to robust biophysical control of ovulation.

## 5 Mechanical signalling from extra-follicular environment

In addition to intra-follicular mechanical interactions, extra-follicular mechanical signals from the ovarian stroma also play a significant role in regulating follicle growth. The stroma is highly complex, consisting of nerves, vasculature, ECM, immune and fibroblast-like cells (Kinnear et al., 2020). In this section, we discuss how the ECM, stromal cells and follicle-follicle interactions may dynamically modulate the follicle’s mechanical environment to orchestrate its development.

### 5.1 Interstitial extracellular matrix

It is well established that the ECM can initiate both mechanical and biochemical signalling cues. Historically, the role of ovarian fibrillar ECM such as collagen, laminin and fibronectin, and their changes during follicular progression were the focus of in-depth characterisation (Berkholtz et al., 2006). Across species, collagen IV is consistently expressed in the basal lamina throughout follicle development (Irving-Rodgers and Rodgers, 2005; Ouni et al., 2020). They were also detected in the BM of atretic follicles (Nakano et al., 2007) and the theca shell (Figure 3A). However, type IV alpha chains 3-6 in the BM decreases after the primary follicle stage (Irving-Rodgers and Rodgers, 2005). Collagen I and VI are expressed throughout the ovary (Woodruff and Shea, 2007; Ouni et al., 2019), with type I mainly located at the ovarian surface epithelium (Woodruff and Shea, 2007). Spatially, decellularized human and bovine ovarian tissues show that the collagen fibres are radially aligned at the cortex and anisotropic at the medulla (Laronda et al., 2015), suggesting a difference in ECM mechanics between the cortex and the medulla, possibly associated with the cortex having a higher stiffness. Recent work on human ovaries also revealed a significant thickening of ECM fibre bundles and increased ECM pore size from prepuberty to reproductive ageing, which is also associated with increased fibre orientation and straightness (Ouni et al., 2021). Similar to collagen, fibronectin was reported to increase in the stroma and theca layer during follicle growth (Woodruff and Shea, 2007). Laminin was also found to increase in the BM along with follicle development (Irving-Rodgers and Rodgers, 2005), with some isoforms present in the ovarian surface epithelium and TC layer (Rodgers et al., 2003).

Apart from fibrillar proteins, a key component of the ECM is hyaluronan or hyaluronic acid (HA), which is an anionic glycosaminoglycan (GAG) matrix with high molecular weight (typically 1,000-8,000 kDa) (Cowman et al., 2015; Monslow et al., 2015). Their ability to trap fluid and swell by osmotic pressure generates mechanical stress to influence the form and function of the tissue microenvironment (Voutouri and Stylianopoulos, 2018). In zebrafish, HA provides anisotropic extracellular stress to guide otic bud morphogenesis (Munjal et al., 2021) or cardiac valve development (Vignes et al., 2022). In murine adenocarcinoma models, administration of hyaluronidase resulted in tumour relaxation, indicating that HA provides tissue compressive resistance (Stylianopoulos et al., 2012; Voutouri and Stylianopoulos, 2018). In bovine and porcine ovaries, HA was localised at the theca layer, stroma, and vasculature (Parkes et al., 2021), similar to our observation in mice (Figure 3B). HA concentrations are known to surge and accumulate in the cumulus cell-oocyte complexes (COC) (Salustri et al., 1999), potentially related to their soft nature (Chen et al., 2016). In contrast to collagen, the roles of HA mechanics in regulating folliculogenesis and ovarian functions are relatively understudied.

There has been recent progress in understanding the spatio-temporal dynamics of interstitial ECM in the context of ovarian ageing, fibrosis, and inflammation. During ovarian ageing, the overall collagen deposition increases while the total HA content decreases, with no significant change in the molecular weights (Amargant et al., 2020). This fibrotic trend was again observed in human samples, indicating that the phenomenon may be conserved across species during ageing (Manuel et al., 2019). A recent study has also shown that this ageing-associated reduction in female reproductive lifespan can be rescued by reversing collagen fibrosis in mouse ovaries (Umehara et al., 2022). It is known that fibrosis mechanically alters the organ by significantly increasing tissue stiffness (Wells, 2013). However, it remains unclear as to how fibrotic ECM remodelling of the ovarian stroma mechanistically modulates follicle development in disease and ageing (Figure 3C).

### 5.3 Stromal cells

Apart from the ECM, the ovarian stroma is also populated with diverse cell types with myriad functions. Amongst the stromal cells, fibroblasts (Sahai et al., 2020) and macrophages (Kim and Nair, 2019) are known to have the capability to remodel ECM. Indeed, there have been extensive reports of ECM remodelling *via* matrix metalloproteinases, plasminogens, and associated enzymes in ovaries (Smith et al., 1999; Curry and Smith, 2006). However, which stromal cells are specifically involved during ECM deposition and degradation is not fully understood.

Macrophages make up the largest sub-population of immune cells in the ovaries (Kinnear et al., 2020), and are known to possess ECM remodelling capabilities which can enhance ovarian cancer progression (Busuttil et al., 2014; Cheng et al., 2019). In aged ovaries, it was reported that there was a shift in macrophage polarisation towards pro-tissue regenerative type (M2 polarisation) (Zhang et al., 2020) which is coherent with the observed inflammatory fibrotic ECM structure in aged ovaries (Briley et al., 2016; Rowley et al., 2020; Lliberos et al., 2021), although the underlying mechanotransduction pathways remain unknown. Fibroblasts, in their highly contractile state, are generally associated with ECM deposition and remodelling (Sahai et al., 2020). While there have been several reports linking cancer-associated fibroblasts with ovarian cancer progression and tumour-ECM remodelling (Kenny et al., 2007; Thuwajit et al., 2018), little is known about how the ovarian fibroblast-like cells modulate the ovarian matrix during folliculogenesis. The study of ovarian fibroblasts is challenging due to a lack of suitable experimental platforms (Quiros et al., 2008). This is further compounded by the fact that fibroblasts lack a clear biomarker, and have been shown to be highly heterogeneous between and within organs (Chang et al., 2002).

### 5.4 Stromal cell-ECM-follicle feedback

While the stromal cells can actively remodel the ECM, ECM biomechanics can also reciprocally impact the stromal cell functions (Figure 3D). It has been shown that macrophage function, polarisation, and migration are influenced by ECM biomechanical and physical properties (McWhorter et al., 2015; Sridharan et al., 2019). For example, macrophages seeded onto stiff polyacrylamide gels were primed towards pro-inflammatory polarisation and exhibit podosome-dependent migration whereas on soft gels, macrophages were anti-inflammatory and showed fast ameboid-like migration (Petersen et al., 2012). Likewise, the ECM remodelling capability of fibroblasts is also dependent on physical cues from the environment–it was observed that high stiffness scaffolds increased fibroblast secretion of matrix metalloproteinase-1 (Petersen et al., 2012). Interestingly, we identified F4/80+ macrophages to be spatially associated with the HA ‘shell’ at the theca layer (Figure 3B), suggesting possible crosstalk between macrophage and HA. Active remodelling of ECM by the stromal cells may lead to changes in the ECM biomechanical properties, which will ultimately impact follicle growth and oocyte quality (Figure 3D). The stromal cells, particularly the macrophages, are known to be associated with atretic follicles across different species (Kasuya, 1995; Best et al., 1996; Petrovská et al., 1996; Gaytán et al., 1998) and in the ovulatory process (Brännström et al., 1993; Takaya et al., 1997), which suggests that the macrophages may play an active role in tissue clearing and wound healing processes during ovarian development and homeostasis. Ultimately, a system-level understanding of the feedback interactions between the stromal cells, ECM, and the follicles will help illuminate the underlying mechanobiological principles regulating ovarian folliculogenesis (Figure 3D).

### 5.5 Follicle-follicle interactions

Another source of mechanical signalling *in vivo* could be the direct contact between the follicles. In a seminal work by Spears *et al.* (Spears et al., 1996), they demonstrated that co-culture of two mouse follicles in contact can lead to follicle dominance, where one follicle invariably grows and becomes dominant while the other one shows arrested growth, as shown in Figure 3E. Notably, the suppressed follicles are not dying, as manual removal of the dominant follicle can induce subsequent growth of the suppressed follicle. In a follow-up paper, the authors further demonstrated that the dominant follicles also render the subordinate follicles more susceptible to lowered FSH concentrations, thereby inducing atresia in these follicles (Baker et al., 2001). These findings on how follicle-follicle contact determines follicle selection and fate may answer the question why only a certain number of follicles undergo size amplification and eventually ovulate during each reproductive cycle (6-7 ovulatory follicles in the case of mouse and only one in human). These findings are physiologically relevant as histological studies revealed that preantral follicles are often found in close contact with each other (Da Silva-Buttkus et al., 2008) (Figure 3F), and preovulatory follicles are found alongside less-developed antral follicles (Baker and SpearsN., 1999). These studies also prompt speculation if differential follicle growth can lead to distinct follicle positioning within the ovary during development. For example, in very young ovaries, the small primordial follicles are often found at the ovarian cortex while the larger growing follicles are found in the inner medulla. Later, as the mice grow past puberty, the ovary is characterised by numerous antral follicles that are found to be close to the ovarian surface, ready for ovulation. Whether such tissue patterning is mediated by follicle-follicle interactions and if these interactions play a similar role in human ovaries are exciting questions for the future.

In the above studies, the authors proposed the existence of a contact-mediated mechanism for follicle dominance and suppression, although the molecular mechanism was not addressed. Others have proposed that the growing follicles may secrete inhibitory signals such as anti-Müllerian hormone (AMH) and activin to maintain primordial or pre-antral follicle dormancy (Durlinger et al., 1999; Mizunuma et al., 1999). Here we propose that in addition to paracrine signalling, mechanical signals, such as the compressive stress exerted by one follicle on another, may provide further inhibitory signals to break the initial size symmetry and trigger differential growth. This corroborates with a recent atomic force microscopy (AFM) study which revealed that the large follicles are the mechanically dominant structures in the ovary that may transmit mechanical stress to the surrounding follicles (Hopkins et al., 2021).

## 6 Approaches to study ovarian mechanobiology

### 6.1 Biomechanical characterisation of ovarian mechanics

The concept of mechanobiology in ovarian biology is relatively new, and biophysical studies to quantify tissue mechanical properties in ovaries remain scarce. To date, AFM remains the primary tool to measure the ovarian biomechanical properties, such as the follicle and stromal stiffness during reproductive ageing and menopause (Amargant et al., 2020; Hopkins et al., 2021; Ouni et al., 2021). Here, the sample is indented with a cantilever attached to an AFM tip of known geometry and the applied force is measured from the bending angle of the cantilever. The surface stiffness is calculated by fitting the force indentation curves to known mechanical models. Of note, while these studies were conducted on different layers of tissues, tissue sectioning itself may potentially release tissue stress and perturb the mechanical state of the ovaries (Stylianopoulos et al., 2012). Micropipette aspiration is another technique that can be used to probe the surface tension of a cell or tissue, based on the Laplace Law (Maître et al., 2016; Chan et al., 2019). Furthermore, by tracking the dynamics of the aspirated ‘tongue’ of the tissue, the tissue viscosity can be extracted (Guevorkian et al., 2010). Another well established technique to infer cellular or tissue tension is laser ablation. Here, UV or femtosecond-pulsed near-infrared lasers are used to cut biological tissues locally. Upon disruption, the speed and direction of recoil of the cell-cell junctions or tissue segment can be used to infer the magnitude and orientation of local cell or tissue tension (Sugimura et al., 2016).

The above-mentioned techniques are mostly confined to measuring the surface mechanics of tissues. To quantify mechanical properties within the tissues, other tools such as magnetic tweezer have been developed. Here, magnetic beads or ferrofluid oil droplets are introduced into tissues, which can be manipulated in the presence of an externally applied magnetic field. If the magnetic properties of the beads or droplets is known, the force-displacement profiles can be used to calculate the local stiffness and viscosity of the tissue (Mongera et al., 2018; Zhu et al., 2020; D’Angelo and Solon, 2020). In recent years, label-free, non-invasive approaches have also been developed to directly ‘image’ the mechanical landscape of tissue interior. For example, Brillouin microscopy has been developed to measure micro-viscoelasticity of cells and tissues with high spatial resolution in 3D (Prevedel et al., 2019). It relies on the interaction and inelastic scattering of monochromatic laser light from thermally driven acoustic phonons at high frequencies. The scattered light spectrum is indicative of the material’s local mechanical properties, as shown by recent work on mouse ovaries and living embryos (Chan et al., 2021; Bevilacqua et al., 2022). Another imaging technique is second harmonic generation microscopy, which probes the tissue composition and the molecular structure of collagen with high sensitivity and specificity. It has been used extensively to study alterations in fibrillar collagens in scar tissues of skin, lung, heart and eyes (Mostaço-Guidolin et al., 2017), as well as in mouse ovaries (Watson et al., 2012; Bochner et al., 2015).

Besides measuring tissue material properties, another important parameter that remains poorly characterised is the mechanical stress within ovaries. To evaluate mechanical stress in tissue interior, several techniques have been developed in recent years. For example, hydrogel-based deformable beads have been developed, which allows ones to quantify the local stress fields in living tissues, as demonstrated in zebrafish and *in vitro* cell aggregates (Dolega et al., 2017; Mohagheghian et al., 2018; Träber et al., 2019; Souchaud et al., 2022). Larger beads have been used to infer the contractile stress exerted by cells. For example, a recent study shows that the compressive stress exerted by contractile cardiomyocytes can be quantified by measuring the change in volume of large gelatin beads of known compressibility, before and after cardiomyocyte enwrapment (He et al., 2021). To quantify tissue pressure in 3D tissues, laser ablation, combined with the tracking of tissue outflow from the abscission site, allows one to compare the mechanical state of compressed tumour aggregates (Barbazan et al., 2021). While many of these techniques have yet to be applied to mammalian ovaries, we foresee that such quantitative studies will yield important insights to advance the field of ovarian biology.

### 6.2 Quantitative imaging of ovarian dynamics

Recent advancement in deep tissue imaging has allowed us to gain insights into follicle dynamics within the ovary. One such tool is light-sheet microscopy. This technique has been increasingly used in the last few years for rapid visualisation of live specimens, particularly for deep tissues where a single sample plane is excited optically by a light sheet and fluorescent images are captured by a camera placed perpendicular to the excitation (Power and Huisken, 2017). This technique allows an enhanced image quality and does not rely on physical sectioning of the tissues. Combined with tissue clearing, light-sheet microscopy has been used to quantify follicle morphometrics in pig ovaries (Lin et al., 2017) and human ovaries that revealed marked architectural remodelling in certain ECM biomarkers at prepubertal, reproductive-age and menopausal stages (Ouni et al., 2022). Another powerful tool for studying follicle development *in vivo* is optical coherence tomography, which allows imaging of ovaries *in situ* and in real time with micron-scale imaging resolution (Wang et al., 2015; Watanabe et al., 2015). Widely used in ophthalmology and endoscopic studies of luminal organ systems (Swanson et al., 1993; Fujimoto et al., 2000), there has been a growing number of work utilising this approach to study reproductive events when combined with intra-vital imaging, such as in mouse ovary and oviduct (Burton et al., 2015), co-operative sperm flow (Wang and Larina, 2018), and oocyte and embryo transport during pre-implantation development (Wang and Larina, 2021).

### 6.3 *Ex vivo* reconstitution

**FIGURE 4:**
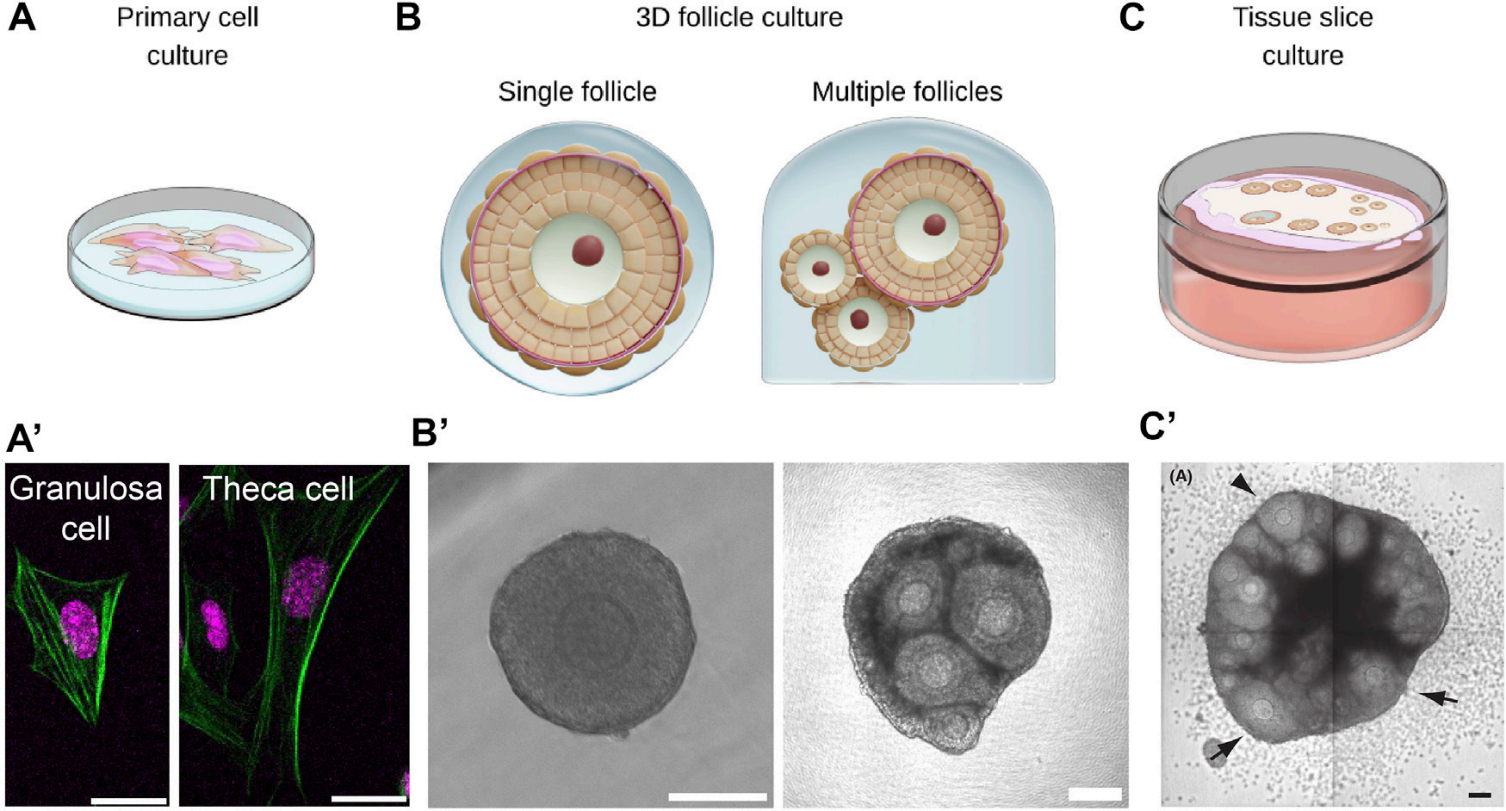
Multiscale *ex vivo* reconstitution approaches are required for integrative understanding of ovarian mechanobiology. (**A,A’)**. Primary cells such as the theca cells and the granulosa cells can be isolated from bulk ovaries for studying single cell mechanics or cell-cell interactions in co-culture systems. Scale bar: 20 μm. **(B,B’)**. Isolated follicles can be cultured in 3D hydrogel for studying morphogenesis and the impact of mechanical confinement on follicle growth. Aggregates of follicles can also be cultured in 3D to study follicle-follicle interactions. Scale bar: 100 μm. **(C,C’)**. Ovarian tissue slices can be cultured *ex vivo* to study collective dynamics of follicles and ovulation during development. Scale bar: 100 μm. Image from (Komatsu and Masubuchi, 2017).

Owing to the multifactorial complexity in the tissue microenvironment, it is challenging to dissect the individual contribution of mechanical signals on follicle growth. Here, *ex vivo* reconstitution of follicles provides a bottom-up approach to understand how the follicle’s mechanical environment impacts its maturation. For example, when cultured in 3D alginate hydrogel (Figure 4B,B′), secondary follicles in the softer matrix showed increased follicle growth, theca development, antrum formation and higher oocyte quality, suggesting that mechanical confinement can regulate follicle development (Xu et al., 2006b; West et al., 2007; West-Farrell et al., 2009). To allow follicle expansion at later stages of development, degradable hydrogel was developed, such as the fibrin-alginate interpenetrating network (Shikanov et al., 2011). Recently, poly(ethylene glycol) (PEG) hydrogels have also been developed to allow better control of matrix stiffness and pore size that promotes optimal follicle growth (Kim et al., 2016). Others have further incorporated mechanical heterogeneities within the gel, where microfluidics was employed to encapsulate follicles within a gel that is made of a soft inner core of collagen and stiff alginate shell (Choi et al., 2014; He, 2017). This was proposed to better mimic the supposedly soft medulla and stiffer cortex of the ovary that are required for follicles to develop to the antrum stage.

In addition to stiffness, ovarian tissues may be viscoelastic and exhibit stress relaxation, which measures how fast the material dissipates its internal stress in response to externally applied deformation. Recent studies demonstrated that hydrogels that exhibit slow relaxation provide greater mechanical confinement to hinder spheroid growth and cell proliferation (Nam et al., 2019; Indana et al., 2021). As discussed before (see Section 5), HA is a major component of ovarian ECM that is highly expressed in the ovarian stroma and TCs. Their ability to trap interstitial fluid and resist compression (Irving-Rodgers and Rodgers, 2005) may generate a slow stress relaxation environment to confine follicle growth. In future, it will be instructive to study follicle growth when cultured in gels of different stress relaxation profiles. Also, how HA remodelling impacts follicle growth and oocyte functions during ovarian ageing will be an exciting topic for future research.

Moving beyond single follicle studies, other works have attempted to study follicle dynamics using ovarian tissue slices (Komatsu and Masubuchi, 2017; Komatsu et al., 2018). By growing tissues on culture inserts, the authors were able to image and quantify pre-antral follicle development, ovulation and atresia (Figure 4C,C′). This approach may provide a good platform to study collective dynamics of follicles, such as differential growth and follicle dominance as discussed in previous sections.

To investigate mechanical interactions between the oocyte and somatic cells, it is noteworthy that there exist protocols for isolating and culturing primary GCs and TCs *in vitro* (Li and Hearn, 2000; Choi et al., 2014; Tian et al., 2015) (Figure 4A,A′). This provides a unique opportunity to probe their biomechanical properties and mechanosensing properties, which is not easily accessible *in vivo*. By adding complexity to such systems, such as TC-follicle or GC-oocyte co-culture systems, one could also gain new insights into how the follicle components mechanically interact with each other.

## 7 Conclusion

With the advancements in mechanobiology and experimental tools, we are now at a unique position to address how mechanical signals act in concert with hormonal signalling to control robust mammalian folliculogenesis. While we have discussed new biophysical techniques that allow us to dissect the intricate mechanical interactions at the intra- and extra-follicular level, *in vivo* perturbations remain challenging given the multiscale complexity of the organ. An attractive approach to address these experimental limitations is theoretical modelling and computational simulations (Johnson et al., 2015; Fischer et al., 2021; Fischer-Holzhausen and Röblitz, 2022), which allow us to tune mechanical parameters and ‘perturb’ the system *in silico* in order to assess functional sufficiency of these mechanical signals in ovarian growth.

Understanding ovarian mechanobiology has profound implications in understanding ovarian ageing, cancer, and disease, which are often associated with impaired tissue mechanics and misregulated mechano-signalling pathways. From the clinical perspective, a ‘mechanical’ understanding of follicular development has important implications in assisted reproductive technology such as *in vitro* fertilisation (IVF) and *in vitro* maturation (IVM). Both approaches generate relatively low success rates, potentially due to the removal of the follicle’s mechanical environment that helps to ensure oocyte health. The design of novel biomaterials to provide appropriate mechanical signals may contribute to future improvement of IVF and IVM and even oocyte rejuvenation. Of note, the ovaries are similar to many organs in terms of tissue architecture and functions, and similar biophysical principles may operate in these diverse systems. Indeed, recent work revealed the role of mechanics in lymphocyte infiltration and stromal remodelling during lymph node expansion (Assen et al., 2022; Horsnell et al., 2022) and mammalian kidney development (Viola et al., 2022).

We end this review by proposing several questions for future research. Does mechanotransduction play a role in regulating oocyte growth? If so, what are the key mechanosensors involved in the process? How are the various mechanical signals integrated across multiple scales to influence follicle maturation? At the tissue level, how are mechanics and hormonal signalling coupled to generate robust follicle patterns in terms of their number, size and positions? Finally, does follicle-follicle interactions play a role in follicle death (atresia) that is known to take place during ovarian development? Recent work revealed that cell-cell fluid exchange can lead to collective cell death and the selection of functional eggs in *C. elegans* during oogenesis (Chartier et al., 2021), suggesting that germ cell fate can be controlled by environmental factors. Ultimately, it will be exciting to explore if the different spatiotemporal dynamics of follicle development in diverse mammalian species are governed by the same underlying mechanobiological principles.
